# 170 Leveraging the Electronic Medical Record to Identify Patients at Risk for Carbapenemase-producing organisms

**DOI:** 10.1017/ash.2026.10571

**Published:** 2026-06-23

**Authors:** Mandy Bodily-Bartrum

**Affiliations:** 1 Vibra Healthcare

## Abstract

**Background:** Central line-associated bloodstream infections (CLABSIs) remain a significant challenge in long-term acute care hospitals (LTACHs), where patients have extended lengths of stay, high device utilization, and complex medical conditions. In 2024, our 11-hospital LTACH system had a CLABSI standardized infection ratio (SIR) of 1.23, performing above (worse than) the national benchmark. A comprehensive, system-wide initiative was needed to reduce preventable infections and improve patient outcomes. **Methods:** In January 2025, we implemented a multi-modal intervention bundle across 11 LTACHs spanning multiple states. Key components included: (1) A standardized mini-root cause analysis (Mini-RCA) process requiring facility infection preventionists to complete a structured review within 48 hours of each CLABSI event, examining insertion and maintenance bundle compliance, contributing factors, and preventability. Findings were shared with frontline staff and reported to each facility's QAPI committee. (2) Beginning August 2025, corporate infection prevention oversight calls were initiated to review events and identify system-wide patterns. (3) In-house vascular access training was expanded to three facilities, enabling nurses to insert PICCs and midlines under ultrasound guidance, reducing reliance on external contractors and empowering staff to evaluate line necessity and step-down opportunities from central lines to less invasive devices. (4) A blood culture stewardship program was implemented in partnership with pharmacy, emphasizing appropriate culturing indications, transitioning from line draws to two peripheral sticks when feasible, and antibiotic stewardship principles. **Results:** Comparing full-year 2024 data to January-November 2025, CLABSI events decreased from 56 to 22 (61% reduction). The system SIR improved from 1.23 to 0.53, representing a 57% improvement and performance significantly better than the national benchmark. Central line days decreased by 8% (40,825 to 37,570), and the standardized utilization ratio (SUR) improved from 0.77 to 0.73. **Conclusions:** Implementation of a multi-modal intervention bundle featuring standardized event review, corporate oversight, enhanced vascular access capabilities, and blood culture stewardship achieved significant and sustained CLABSI reduction across a geographically dispersed LTACH system. The Mini-RCA process created accountability and rapid feedback loops, while in-house line placement empowered clinical staff to critically evaluate device necessity. This approach demonstrates that meaningful infection prevention improvements are achievable in the challenging LTACH setting through systematic, multi-level interventions.

